# How Basic Scientists Help the Pharmaceutical Industry Market Drugs

**DOI:** 10.1371/journal.pbio.1001716

**Published:** 2013-11-19

**Authors:** Adriane Fugh-Berman

**Affiliations:** Georgetown University Medical Center, Washington, D.C., United States of America

## Abstract

Basic science research is not immune to the influence of pharmaceutical and industrial partners. This Perspective by Adriane Fugh-Bermann explains why scientists should be aware of the potential competing interests posed by practices such as selective publication and ghost-writing.

In the 1970s, a Baltimore city senator who also owned a tavern backed legislation that helped his business. Accused of having a conflict of interest, Joseph J. Staszak, responded, “What conflict of interest? How does this conflict with my interest?” [1].

According to the Institute of Medicine, a conflict of interest is “a set of circumstances that creates a risk that professional judgment or actions regarding a primary interest will be unduly influenced by a secondary influence” [2]. More simply, conflicts of interest may be seen as circumstances in which “individuals' professional responsibilities diverge from their personal interests (or when different professional responsibilities clash)” [3].

In biomedicine, discourse on conflicts of interest (also called competing interests) has focused on relationships between industry and physicians or clinical researchers. However, basic scientists are not immune to industry influence on research and publications, and may be important to industry in the production and dissemination of marketing messages.

## Depending on Industry

In 2007, industry was the largest funder of biomedical research, paying for nearly twice as much research (58%) as the federal government (33%) [4]. Most of this funding goes to clinical research; the share of spending by pharmaceutical and device industry on preclinical research has decreased from about half (55%) in 1998 to a quarter (25%) in 2010 [5]. A 2007 survey of 3,080 academic life science researchers found that half (53%) have some form of relationship with industry [6]. Among the 1,663 research faculty at academic medical centers, 42% of basic scientists had a relationship with industry. This number was similar to health services researchers/clinical epidemiologists (40%), but less than clinical researchers (67%) translational researchers (61%), or “multimodal” researchers (71%) [7]. At the 50 universities that received the most NIH research funding, 43% of 2,167 life science researchers reported receiving a research-related gift in the late 1990s [8]. Gifts included biomaterials (24% of respondents), discretionary funds (15%), equipment (11%), travel funds to professional meetings (11%), student support (9%), and other (3%).

Researchers were aware that something was expected in return for the gift. Sponsor expectations that the gift be used for its intended purpose and not be re-gifted, and that the sponsor be acknowledged in publications, are certainly reasonable. Disturbingly, however, about a third (32%) of gift recipients reported that the funder wanted prepublication review of any articles or reports stemming from the use of the gift. This expectation was higher for gifts of biomaterials: 40% of respondents reported that the firm wanted to receive prepublication review of articles or reports. Also, 44% of firms wanted assurances that the biomaterial was not to be used for applications that competed with company products [8].

## Industry Funding Affects Results

In clinical research, investigators who receive industry funding are more likely to publish results that favor a sponsor's marketing goals than are investigators who do not receive industry funding. The Cochrane Collaboration, renowned for creating and publishing high-quality systematic reviews, analyzed 48 clinical studies, systematic reviews, and meta-analyses that compared results from studies of drugs or medical devices based on sponsorship. This systematic review found that industry-sponsored studies, compared to non-industry-sponsored studies, were more likely to report favorable efficacy results for drugs or medical devices; less likely to find harms; and more likely to conclude that a therapy was beneficial [9].

Systematic reviews are important because they utilize a scientifically reliable method that identifies, evaluates, and synthesizes evidence that meets pre-specified criteria and use explicit methods to avoid bias in selecting studies for review. Conventional literature reviews may pick and choose what to include, and while some are useful, non-systematic reviews should be viewed as opinion pieces.

The Cochrane review focused on clinical trials, and less is known about the effects of industry funding on basic science research. What information exists, however, is not reassuring.

## Design and Reporting Biases

Positive results for therapeutics in animal studies are often not confirmed in clinical studies. Species differences certainly play a role, but faulty experimental design, reporting bias, analytic bias, and publication bias also may be important.

A recent analysis of 4,445 animal studies in 160 meta-analyses of neurological drug candidates found that far too many studies (1,719) had a “positive” result, when only about half that number (919 studies) would have been expected to be positive [10]. Design and reporting biases are the most likely explanation. More than two-thirds (70%) of the meta-analyses found a statistically significant summary effect; of these, the vast majority – 108 of 112 studies –favored the therapeutic agent, while only four studies favored controls. An analysis limited to studies with more than 500 animals that found significant positive effects of treatment and showed no suggestion of bias identified only eight treatments—5% of the 160 treatments—that should have graduated to testing in humans [10].

Cell culture studies may also suffer from design or reporting bias. Let's take the example of erythropoetin-stimulating agents (ESAs), which were licensed in the early 1990s for treating anemia in cancer patients and became widely used. Early published clinical studies and meta-analyses appeared to show a benefit of ESAs on mortality [11]. It is now known, however, that ESAs actually increase thromboembolic events, cardiovascular events, and overall mortality in cancer patients [12]; disturbingly, knowledge of this risk was delayed by a decade because of publication and reporting biases [11].

Long before an increased mortality risk in humans was established, however, questions about potential adverse effects of ESAs had been raised. Basic science studies were conducted to address the question of whether ESAs fostered malignant growth. An analysis of published basic science research found that researchers funded by ESA manufacturers were far less likely than non-industry-funded investigators to report that ESAs promoted tumor growth [13]. For example, 94% of studies by non-industry-funded researchers (32 of 34 studies) found that erythropoetin had the potential to promote malignancy by inducing signaling events. None of the ten researchers funded or employed by ESA manufacturers reported EPO-induced signaling events. While 57% (24 of 42 studies) of non-industry-funded investigators found erythropoietin-induced changes in cellular function, none of the seven researchers paid by ESA manufacturers found these malignancy-promoting effects.

The majority of researchers (57%) without ties to ESA manufacturers concluded that ESAs were linked to increased malignant potential, but not one researcher funded by ESA makers drew that conclusion. Several industry-paid researchers actually concluded that erythropoietin had potential antitumor effects [13].

In other words, industry-supported basic science researchers found industry-friendly results. Although the question of whether ESAs promote tumor progression in humans remains open, the contribution of industry-funded researchers to reassuring clinicians and policymakers regarding risks of ESAs remains concerning.

## Selective Publication

Selective presentations and publications are important tactics for industry. Industry relies on abstracts and posters to convey marketing messages at scientific meetings, because abstracts and posters are usually not peer-reviewed and can be easily altered up to the time of presentation. Posters and abstracts are often used for preclinical studies, case reports, or preliminary results of clinical trials. Promising preliminary results might be presented as a poster, and the results may be publicized, but if the final results of the study do not support commercial goals, the full study may never be published - or may be buried in an obscure, low-impact journal. In either case, scientists may have a positive impression of a therapy from a poster, and never learn that the therapy failed to show efficacy in the final study.

The majority of meeting abstracts and posters are never published. Posters and abstracts with positive results are far more likely to be published than negative studies [14],[15]. A 2007 Cochrane systematic review of 79 reports found that less than half (44%) of 29,729 abstracts presented at scientific meeting were subsequently published as full studies [14]. More recent studies have also found low publication rates; 42% of posters at three annual nephrology meetings remained unpublished 4.5 years later [16]; the overall rate of publication of abstracts and posters at a neurosurgery meeting was 32% (1,243 of 3,827 abstracts) [17]; and only 62% of abstracts presented at meeting on drug addiction were published [15]. It should be noted that these studies may not have picked up data from posters that was incorporated into other articles rather than being published as freestanding articles.

Selective publication of studies that favor a sponsor's drug has obvious commercial benefits. According to the former pharmaceutical executive who shared his perspective on condition of anonymity:

*“It is to industry's advantage to selectively support particular researchers whose point of view supports marketing goals, and to encourage selective publication of articles.”*

—Former pharmaceutical executive, personal communication

Although many universities frown on researchers signing agreements that give funders the right to suppress the publication of findings, school policies lack uniformity regarding publishing. Researchers cannot be forced to publish studies. Writing up studies requires time and effort, and researchers may be less motivated to publish negative data, especially when it is perceived that negative studies have less impact than positive studies.

Anecdotally, researchers say that good journals won't publish negative studies, but journals cannot publish what is not submitted. A study of papers presented at a gastroenterology conference found that 76% (156 of 206) of papers that were never published were never submitted [18].

Perhaps because negative outcomes from industry-funded studies are less likely to be submitted for publication, industry-funded clinical trials overall are less likely to be published. An analysis of 546 drug trials listed in ClinicalTrials.gov found that within two years of study completion, about a third of studies that received full (32%) or partial (39%) industry support were published. In contrast, more than half (54%) of trials funded by government, and 56% of trials funded entirely through nonprofit/nonfederal funds, were published [19].

Less information is available about basic science studies, but there is reason to be concerned. A key analysis of 16 systematic reviews of experimental animal studies of acute ischemic stroke found that 98% (515 of 525) of unique publications reported significant effects of treatment on infarct volume [20]. It's impossible to say exactly how many negative studies went missing, but using a statistical technique called trim-and-fill to account for missing data, it was estimated that 214 experiments were conducted but not reported; the non-publication rate was estimated to be 14%.

The authors estimate that publication bias may have accounted for about a third of the efficacy reported in systematic reviews [20]. In other words, the absence of negative studies may result in systematic reviews erroneously concluding that a therapy is more beneficial than it actually is.

Researchers may not submit papers for publication for many valid reasons. In contrast to clinical research, in which the goal is often to determine whether a specific drug is safe or effective in a specific population, the goal in much of basic science is to define mechanisms and advance understanding of biological processes. Full publication of negative results may be seen as less interesting by basic science researchers. Faculty mentors may encourage students to present work as an abstract as an outlet for disseminating information and recognition of a student's efforts, but without the expectation that a full paper will be written.

The question of whether keeping industry sponsors happy affects publishing decisions has important ethical implications. An industry funder provides help, encouragement, and approbation for publishing positive studies. There may be little incentive to preserve the integrity of the medical literature when the unspoken threat of funding withdrawal hangs over a researcher who insists on publishing negative studies.

Nonetheless, research faculty should teach students that there is an ethical obligation to publish negative studies, and should model this behavior. From a practical standpoint, there is little justification for the belief that journals will only publish positive studies. *PLOS ONE* and numerous other journals have pledged to publish high-quality research with negative results (see Table 1).

**Table 1:** Examples of journals that publish negative results.

Journal Name	URL
*PLOS ONE*	http://www.plosone.org/static/publication.action
*The All-Results Journals: Chem*	http://www.arjournals.com/ojs/
*The All-Results Journals: Nano*	http://www.arjournals.com/ojs/
*The All-Results Journals: Biol*	http://www.arjournals.com/ojs/
*The All-Results Journals: Phys*	http://www.arjournals.com/ojs/
*Journal of Negative Results in Biomedicine*	http://www.jnrbm.com/
*Journal of Negative Results in Ecology and Evolutionary Biology*	http://www.jnr-eeb.org/index.php/jnr
*Journal of Pharmaceutical Negative Results*	http://www.pnrjournal.com/
*Journal of Interesting Negative Results in Natural Language Processing and Machine Learning*	http://jinr.org/

## The Use of Basic Science for Marketing

Besides being used to mitigate concerns about adverse effects, preclinical studies are used to promote a marketed drug for a condition for which efficacy in humans has not yet been shown or has been disproven. According to a former pharmaceutical executive who wishes to remain anonymous:

*“The work of basic scientists is used for indirect and sometimes direct marketing to highlight a therapy's mechanism of action, to suggest surrogate markers of safety and efficacy, and to differentiate a product from competitors' based on these findings. Preclinical work is also used to support clinical messages in academic presentations with a more ‘scholarly’ understanding of new science.”*

—Former pharmaceutical executive, personal communication

Promotion of a drug may start 7–10 years before it is submitted for regulatory approval [21], when it is still in animal studies, so preclinical studies may be used to create “buzz” about a new drug years before it arrives on the market. Bias in the representation of both basic science and clinical studies has been found in certain review articles of rimonabant, an endocannabinoid antagonist, before the drug was considered for regulatory approval [22]. In Europe, rimonabant was approved for treating obesity in 2006, then removed from the market in 2008 following reports of suicide and other adverse psychiatric effects. The US FDA did not approve rimonabant.

Once a drug is on the market, it can be prescribed “off-label” – that is, for any condition other than that for which the drug was approved. Although it is legal for physicians and other prescribers to prescribe a drug off-label, it is illegal for pharmaceutical companies to promote drugs off-label. Off-label use is common, accounting for about one in five prescriptions [23]. It is unknown how much off-label use is due to promotion.

Pharmaceutical companies use paid speakers, consultants, and researchers to promote off-label use [24]. For example, to expand the market for the anti-seizure drug Neurontin (gabapentin), Parke-Davis employees recruited physicians to “expand the speaker base—identify and train strong Neurontin advocates and users to speak locally for Neurontin.” The company also provided research grants to “key influencers” and organized a named lecture series featuring neurology department chairs and clinical program directors to improve “public relations within the neurology community, etc., as well as [to impact] the volume of Neurontin new prescriptions.” To promote off-label prescribing for neuropathic pain and bipolar disorder, Parke-Davis directly and indirectly funded many continuing educational programs and used a so-called publication strategy “to disseminate the information as widely as possible through the world's medical literature” [25].

Similar tactics were used to persuade prescribers and patients that hormones prevented disease in postmenopausal women. Physicians paid by Wyeth, the manufacturer of the bestselling menopausal hormone therapy Prempro (conjugated equine estrogens and medroxyprogesterone acetate) spoke at many medical meetings, promoting hormones off-label to prevent cardiovascular disease and prevent dementia in menopausal women. No randomized clinical trials with disease endpoints supported this use, so speakers invoked observational studies, studies with surrogate endpoints (i.e. cholesterol-lowering), experimental animal studies, and even cell culture studies.

At several medical meetings in the early 2000s, I observed speakers paid by Wyeth showing an industry slide that compared brain cells grown in media with and without estrogen. Brain cells grown in estrogen-free media were shriveled and dying, a pathetic counterpoint to the vigorous confluence of cells grown in estrogen-containing media. In living women's brains, however, estrogen had adverse effects. The Women's Health Initiative Memory Study (WHIMS), which studied 7,479 women over 65, found that hormone therapy increased the risk of dementia and cognitive impairment, decreased global cognitive function, and was associated with more brain atrophy [26]. Nonetheless, a 2010 New York Times article promoting the use of hormones for brain health quoted several Wyeth-linked researchers, including two basic scientists, Thomas Clarkson and Roberta Diaz Brinton. Their conflicts of interest were not disclosed [27],[28].

WHIMS was part of the Women's Health Initiative (WHI), a large, long-term, NIH-funded, definitive randomized controlled trial of menopausal hormone therapy in 26,000 women 50–79 years of age, that showed that the risks of menopausal hormone therapy outweighed benefits. Although hormones had been marketed to decrease the risk of cardiovascular disease, the WHI found that hormones were ineffective in preventing heart attacks in healthy women (if anything, hormones increased the risk) and increased the risk of stroke and blood clots [29].

Wyeth-linked experts fanned out to criticize the WHI, attempting to counter the findings by, among other tactics, touting experimental animal studies that showed that estrogen appeared to prevent manifestations of vascular damage in animals fed a high-fat diet [21]. Ghostwritten articles invoked basic science studies [30]. For example, a 2005 document disclosed in litigation from Wyeth's ghostwriting firm, DesignWrite, outlines a planned article, “The Atherosclerotic Process and the Impact of Estrogens” [31],[32] (the title was later changed to “The Impact of Timing of Initiation of Therapy on the Cardiovascular Effects of Postmenopausal Hormone Replacement Therapy” [33]) that would use basic science studies and observational studies to argue that the WHI was a flawed study, and that hormones given to younger women could still have potential disease prevention benefits.

## Ghostwriting and Ghost-Management

Companies have paid billions of dollars in fines for off-label promotion, often using company-generated research, company-paid speakers, and ghostwritten articles to imply clinical benefits in the absence of clinical trials (or the presence of negative trials); fines have also been imposed for suppressing risks or misleading clinicians about risks [34]. For example, in June 2012, GlaxoSmithKline agreed to pay a record-breaking $3 billion to the US government to settle allegations that it failed to report adverse events related to Avandia (rosiglitazone, a diabetes drug) and that it promoted the antidepressants Wellbutrin (buproprion) and Paxil (paroxetine) off-label [35]. The plea agreement describes how GSK hired contractors to ghostwrite “false and misleading” articles that claimed, for example, that the safety and efficacy of Paxil for adolescent depression had been demonstrated despite the fact that the study cited failed to demonstrate efficacy in its primary and secondary endpoints. This publication also minimized adverse effects.

Many pharmaceutical companies use medical education and communication companies (MECCs) to recruit academic physicians and scientists to “author” publications crafted by industry [4]. Articles may be ghostwritten by a medical writer [36]–[38]. Authors who actually write their own articles may still submit to “ghost-management” [37], allowing a company to provide statistical analysis or “editorial assistance” (often an industry code word for ghostwriting), either of which provides a company the opportunity to insert marketing messages into an article.

Ghostwriting has been used to promote Zyprexa (olanzapine) [39], Paxil (paroxetine), “Fen-phen” (fenfluramine and phentermine, used for weight loss), Neurontin (gabapentin, approved for seizures), Vioxx (rofecoxib, an analgesic), and Zoloft (sertraline, an antidepressant) [25]. Undoubtedly, many other drugs are promoted by ghostwriting.

The extent to which basic scientists participate in ghostwritten articles is unknown. Academic medical centers are starting to pay attention to the fact that academic faculty are putting their names on articles they have not written, but in 2010 only 13 (26%) of the top 50 academic medical centers in the US had policies in place that prohibit participation in medical ghostwriting [40]. While these policies are laudable, no researcher in the US has yet been sanctioned for ghostwriting [41].

Even if a researcher does not allow a sponsor to ghostwrite an article, industry review of articles by a sponsor may result in the insertion of subtle marketing messages that researchers may not recognize as advertisements. Marketing messages may not mention the targeted drug; for example, marketing messages may claim that a targeted disease is underdiagnosed, that a mechanism of action is particularly exciting, that a class of drugs has unique benefits, or that a competing drug has significant drawbacks. Marketing messages are disseminated in research studies, case reports, reviews, commentaries, and letters, as well as in presentations and posters at medical meetings [24].

## Conclusion

Biomedical researchers depend on industry. Accepting a sponsor's gifts or statistical or editorial “assistance” creates an opportunity for results to be suppressed or spun to advantage a targeted drug or disadvantage competing therapies. Participating in ghostwriting or ghost-management of publications or posters is ethically unacceptable, as is accepting sponsor suggestions on whether or not to publish, present, bury, or selectively report specific studies.

The underreporting of negative results and misrepresentation of reported results distorts the biomedical literature, makes therapies look better than they are, can mislead both researchers and clinicians, and may have adverse effects on public health. It goes without saying that scientists should accurately report and analyze studies, but publishing negative data should also be considered an obligation to the scientific community. Negative studies are crucial to assessing benefits and risks of therapies and to determining whether further research is indicated.

As the former pharmaceutical executive puts it:

*“Industry is a major funder of basic science research, and academic/industry partnerships and public/private sector partnerships have become common. These partnerships may provide incentive for academic researchers to do work of value to industry partners and to help demonstrate that value over the drug development process. The potential for disruption of the academic mission is obvious.”*

—Former pharmaceutical executive, personal communication

Clinicians and academic medical centers have been grappling with the implications of industry influence on clinical practice, but the issue has not been discussed widely within basic science. It is time for basic scientists to begin a difficult conversation regarding the ethical and scientific hazards of working with industry.
